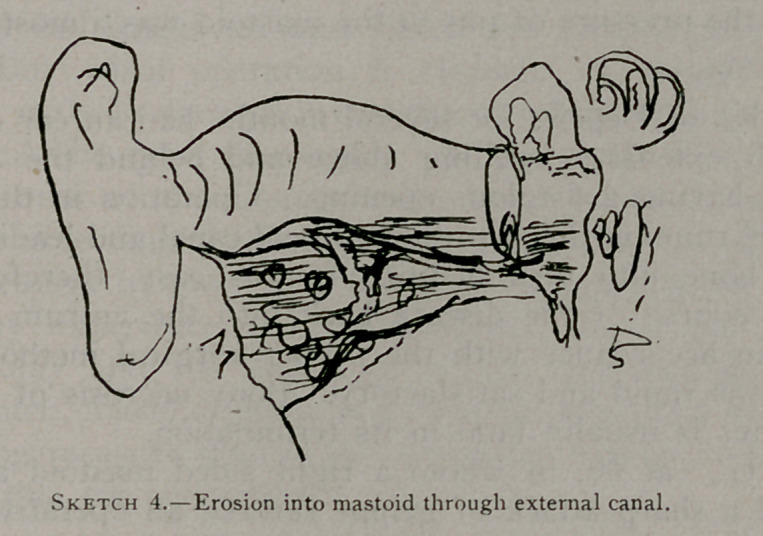# Modern Management of Inflammation of the Middle-Ear and Contiguous Structures

**Published:** 1904-03

**Authors:** F. Park Lewis

**Affiliations:** Buffalo, N. Y.


### Modern Management of Inflammation of the Middle-Ear and Contiguous Structures.

BY F. PARK LEWIS, M. D., Buffalo, N. Y.

THE anatomical relationship of the middle-ear and its adnexa are of first importance, primarily because of their nearness to vital structures, and secondarily because the destruction of any of its essential parts means permanent impairment of an important special sense. The contracted space occupied by the tympanum allows very little room for accumulated products of inflammation as the capacity of the normal cavity would ordinarily contain less than half a dram. The only normal outlet and inlet is the eustachian tube which is designed as an air conductor, and does not readily permit the passage of a dense fluid; there are, however, openings connecting the air cells of the mastoid antrum and passages for the bloodvessels extending into the brain structure and the vascular supply.

The structures within the drum include on its inner surface the bony channel in which is enclosed the facial nerve, and the semicircular canals protecting the delicate tissues of the nervous structure of the inner ear within the skull; and immediately under the mastoid cells is the lateral sinus, but the anatomy is so inconstant that its exact location may never be exactly pre-determined A thin shell of bone separates the attic of the tympanum from the spheno-temporal lobe of the brain.

It will be readily understood, therefore, that the accumulation of pus in this narrow space, with vitally important structures on every side, demands immediate and skilful treatment with the possible alternative of consequences most disastrous. The tympanic membrane is a dense, leathery structure; in a condition of health is capable of supporting great pressure, and fortunately it is exceedingly sensitive.

An acute inflammation of the middle-ear is, therefore, manifested by that great *conservator naturis* warning of coming trouble, great pain; a simple inflammation shows at first under proper illumination red lines running along the handle of the malleus This increases if uncontrolled until the whole membrane becomes rosy red; a secretion is poured out in the little drum cavity, serous at first, soon becoming purulent, and then if the drum membrane is examined through a speculum it is found that its curvature has been altered. It is now swollen outward from the pressure from within, and the pain which neither local adjuvants nor internal anodynes will wholly control has reached its acme, and rupture of the membrane is imminent.

There is but one important procedure at this point. The drum must be opened, a clean incision extending down to its base being

SKETCH 1.—Method of opening membranum tympani; after Dench.

made by an exceedingly sharp and narrow needle-pointed knife. The relief is often immediate. An outlet has been found for the pent-up secretion, and the cleanly divided edges are left in the best possible position for subsequent repair. The resistance of the strong drumhead, should this not be done, may lead in exceptional cases to the diversion of the pus to other channels, and involvement of the mastoid may follow. This is especially likely to be true in young children, where the bony connections are less firmly attached. The following unusual case is illustrative of this point:

A BEZOLD MASTOID CASE.

An Italian child fourteen months old was brought to my clinic at the Charity Eye, Ear and Throat Hospital, and seen by Dr. J. J. Finerty, by whom my attention was called to red, painful swellings behind the ears. An examination of the membranæ

tympani showed them to be dull and pale. There was little change in contour. The child, however, had swollen pus glands on the neck, and it was so evident that the origin of the trouble was the middle-ear that both drum heads were incised when a plentiful discharge of pus followed. Under treatment of the middle-ear and repeated incisions of the drum heads as they closed from time to time, the mastoid swellings gradually disappeared, and when, finally, after about four weeks the discharge had wholly ceased, both tympani and mastoids were left in a perfectly normal condition.

If an acute middle-ear inflammation can be treated early enough, incision of the drum may be avoided, but the ordinary measures recommended in the books are not effective. While sometimes useful, local depletion has usually but little effect on the deeper congestion; in the earlier stages cold applications or antiphlogistine may be helpful, but the preparations of laudanum or other anodynes serve but to mask the real condition.

The local treatment which I have found of greatest value in acute inflammation before pus has formed is the following:

| | | |
|---|---|---|
| ℞ | Cocaine hyd. | 1 |
| | Adrenalin chloride | 2 |
| | Glycerinæ | 4 |
| | Aquæ dist. | 30 |

Sig.—Drop in the ear warm, every half hour or oftener if necessary.

The adrenalin constricts the vessels, and helps in the absorption of the cocaine, which of itself is not taken up freely by the dermal drum surface. The glycerine causes a serous transudation, still further relieving the congested vessels and the combination which is based upon the known action of the drugs will give relief from pain almost immediately when other medicaments seem to be wholly inert.

The treatment of chronic discharges is again a serious matter. Whether or not we are to have a chronic flux will depend in no small degree on our treatment of the primary inflammation. If a free opening extending to the bottom of the tympanum be made in the membrane and the ear be cleaned with a mild antiseptic, such as borolyptol, the cessation of the discharge will probably be followed by a healing of the wound and a complete restoration of the integrity of the membrane. If, on the other hand, the incision is small or the membrane be allowed to rupture from the pressure of the contained secretion, the wound is often a jagged tear, the discharge erodes its edges and shortly a tympanic opening results which from its nature will prove difficult if not impossible to close. The reasons for the difficulties met in controlling a chronic suppuration from the middle-ear are due to its anatomical form, and a failure to bear this in mind when its treatment is undertaken. The form of the external aural canal, it will be remembered, is that of a curve. The highest portion is usually in the middle. A perforation in the drumhead is often not quite at the bottom, and even if it is at the lowest part of the canal the tympanum sinks a little below its edge. The subjoined sketch will show it in effect.

If now a chronic inflammation involves the secreting surface of the tympanic cavity the mucus discharge settling in the bottom of the warm, moist nest, forms a perfect culture medium for the various infectious germs that readily gain entrance through

SKETCH 2.—Pus filling middle-ear to level of perforation.

the perforated membrane. If, as is quite common, syringing be employed, either by the patient or the physician, under usual conditions a little lake is left behind, and this very soon becomes alive with the various cocci that have not been destroyed, and that grow and multiply even more rapidly than before.

It is, therefore, surprising in view of this palpable and self-evident fact that so great an otologist as Bezold, in a recent article in the Archives of Otology, translated by Dr. Arnold Knapp without comment, should decry the most reasonable and efficient method of treatment yet devised, i. e., the wide siphon drainage. In this paper he insists that the gauze becomes saturated and offensive, and by its retention acts as a septic poultice, increasing the condition which it is designed to relieve. This would, of course, be true if the very essence of this method were not in the frequent replacement of the gauze. Its method of action can be more graphically shown by a sketch than by a description.

If the gauze be applied in this manner after thorough antiseptic cleansing of the external and middle-ear, followed by drying with absorbent cotton, the pus is drawn from the middle-ear, which is left in the best possible condition for normal healing. Every detail, however, must be carefully considered. If a granulation exist it must be dexterously removed by cautery, forceps or snare. For preliminary cleansing nothing is more efficient than menthoxol or camphoroxol 1-to-10, followed by lysol 10 to 15 drops in a bowl of warm water. This may be used carefully with a syringe if the discharge is profuse and if a complete drying follow.

The most effective gauze to be used is iodoform, but if one would avoid the disagreeable odor, perhaps equally effective and

SKETCH 3.—Antiseptic gauze in position to siphon fluid from middle-ear.

far pleasanter to use is the bismuth formic-iodide gauze prepared by the Mulford Co. The efficiency of this method is so great that case after case of chronic otorrhea has completely recovered under its employment in my hands. As a local measure in acute suppurative cases threatening mastoid involvement and even when the mastoid had been markedly affected, the newer silver salts have had a most pronounced effect.

### ABORTING ACUTE MASTOIDITIS.

One hesitates to speak of the possibility of aborting acute mastoiditis very much as one is reluctant to urge any measures looking to the aborting of glaucoma or appendicitis, because of the fear that those who are not judicious may carry conservatism beyond the point of safety and court disaster. The otologist, however, who watches the blood, the temperature and the patient will know when it is no longer wise to wait and none other should take the responsibility of caring for a patient with a swollen mastoid or an angry middle-ear. The following two cases in this light are instructive:

A ten year old girl with adenoids was taken with acute inflammation of the middle-ear while being treated for measles by Dr. Wm. H. Marcy. The drum membrane had broken and the discharge was profuse before I was called. Suddenly the temperature, which had been only slightly elevated ran up to 103 degrees and the mastoid became painful. The ear was kept cleansed and then filled with a 25 per cent. solution of protargol; the discharge gradually decreased and the temperature dropped one degree daily until by the time that she was well enough to leave the house, in about two weeks, the ear was fully restored to its normal condition.

Of course, I removed the adenoids at an early date. So important an element in the production of middle-ear disease are adenoids, that it is frequently impossible to cure the former until we have secured suitable ventilation of the cavity by the removal of the pharyngeal obstructions, and it must be further borne in mind, that when children suffer from repeated earache, an obvious or concealed lymphoid hypertrophy may be looked for with every expectation that it will be found.

The second case was one of greater interest for two reasons: first, the rapidity with which resolution followed active measures; and, second, a peculiar feature involved in the treatment. A purulent otitis had been for two months under the care of a skilled otologist when the mastoid became involved. The membranum tympani had been freely opened but the pus poured from the meatus almost as rapidly as it could be removed. The neck was so swollen and stiff that the head could with difficulty be turned. The blood test showed abundant leucocytosis, and the patient, a man of 35, was exceedingly ill. It seemed impossible that a mastoidectomy could be avoided, but as it was late in the day he was sent to the hospital and immediate active treatment instituted.

The ear was cleansed in the manner previously described, and the meatus filled with a 25 per cent. solution of protargol and gently worked into the deeper tissues by slight pressure on the tragus. In the morning he was so much improved that the treatment was continued. In six days he was discharged with a healed drum head and practically well.

In addition to the local measures the important element in his treatment was the almost total withdrawal of all food. During the six days, he had three times daily, only 100 c.c. of cold milk, so that he took during the six days but two quarts of milk altogether. Water he was allowed ad libitum, but no other nourishment of any character whatever. At the time of his dismissal he was in better physical condition than at any time during the previous two months.

The effect of limiting the food supply in this case was so marked that it will certainly be worthy of careful study in all cases in which we have elevated temperature as a result of a retention of toxic products.

When, however, we have pus formation in the mastoid as a result of chronic disease of the middle-ear, with obstruction of the outlet or percolating below the coil of the tympanic opening operative intervention may not longer be postponed. A case which was under my care many years ago in which bony necrosis followed chronic suppurative inflammation of the middle-ear with pus absorption without mastoid involvement, leading to absorption of pus products into the cerebral circulation with sinus thrombosis and death, impressed me profoundly with the danger which always accompanies bone disease in this locality. At that time, the classic works of Schwartze and Stacke, and

SKETCH 4.—Erosion into mastoid through external canal.

Macewen had not been given to the world and operative measures were postponed too long, hence when undertaken were not directed as they would be by our later and fuller knowledge of the probable sequence of events.

A school of German otologists adopts the rule formulated by Bezold that "acute purulent otitis which has existed for more than two months even without complications, is sufficient indication for me to operate" (by opening the mastoid); while it is frequently true that mastoidectomy becomes in such cases ultimately necessary, the method of treatment which I have outlined, especially the use of siphon drainage and protargol of proper strength worked into the deeper portions of the ear after a sufficient opening has been secured in the drum head, has made this with me, far from the general law, but rather the one of many exceptions. At the same time the danger of postponing too long, operative intervention must not be overlooked. The following cases will illustrate in brief the course which such cases may take:

Mrs M., a bride of two months, 25 years of age, had been suffering great pain in ear, profuse discharge, little soreness over the mastoid, free opening in tympanic membrane. In this case persistent treatment failed to control the discharge or relieve the pain.

After two months suffering she was sent to the hospital, where subnormal temperature, faintness, and nausea increased anxiety as to her condition. She then was found to be pregnant.

Under chloroform anesthesia I opened the mastoid, which was found filled with pus, the cells of the antrum had broken down and the cavity was filled with debris. This was thoroughly cureted, and left clean and aseptic; she made a normal and complete recovery.[1].

Under like circumstances I should not delay operation so long, as the pressure of pus in the mastoid was almost a foregone conclusion.

Mr. K., of Depew, for several months had an ear discharging pus with extensive swelling above and behind the auricle, the swelling having a fistulous opening. Operation in this disclosed a channel running down to the external canal and leading through carious bone into the antrum. It was easy, therefore, to follow the course of the disease back into the antrum which was opened in accordance with the proper surgical methods, and recovery was rapid and satisfactory. Bony necrosis of the antrum in diabetes is usually fatal in its termination.

Mr. G., at 60, in whom a right sided mastoid abscess had followed a sharp attack of grippe refused all operative measures until it became evident even to him that surgical intervention was necessary. An exposure of the bone developed necrosis of the entire mastoid portion of the right temporal bone, and the whole of the right parietal. The operation was long and protracted; meningeal inflammation followed resulting in death.

The necessity of a correct analysis of symptoms is shown by the following case:

Miss R., from Pittsburg, while travelling took a severe influenza involving both ears. Acute suppurative inflammation necessitated repeated tympanic punctures, but notwithstanding every possible effort the drum head grew soggy, the external canal exquisitely tender, and the mastoid sore. Temperature subnormal with occasional elevations most suggestive of sepsis; she felt sick and weak. At no time, however, did a mastoidectomy seem warranted. One afternoon after an especially poor report in the morning, I was hastily summoned by a telephonic message that my patient was numb on one side, had vomited and was not

1. A curious feature was an indentation over the left mastoid of the child corresponding to the site of operation in the mother.

seeing clearly; I hastened to her bedside after sending a hurried message to the hospital to have the surgery immediately prepared for an operation only to find her perfectly comfortable, the numbness having disappeared and the nausea having been relieved by vomiting; she then told me that she had frequently had like attacks before her ears had been affected. She was suffering from an attack of scintillating scotoma, which was evidently wholly disconnected with the disease in her ears. She recovered satisfactorily and completely with perfect hearing without any further operative intervention whatever.

The conclusions to which such cases lead us, are that every otitis demands careful individual attention and close clinical observation. Judicious local treatment will in a large number of cases render unnecessary operations which are never simple, which may be dangerous, and which are always protracted in healing. But when operation is required, it should be done sufficiently early to prevent involvements which may prove fatal, and it should be done thoroughly. It has to do with the most vital structures of the human body, and abnormalities of formation are exceedingly common. It should never be undertaken, therefore, except by those who either on the cadaver, by observation, or by experience have familiarised themselves with the bony anatomy and the technique of the operation and in the hands of such, mastoidectomy is an operation no less valuable as a life saving measure than is the somewhat analogous abdominal operation, that for appendicitis.

454 Franklin Street.

## Figures and Tables

**Sketch 1. f1:**
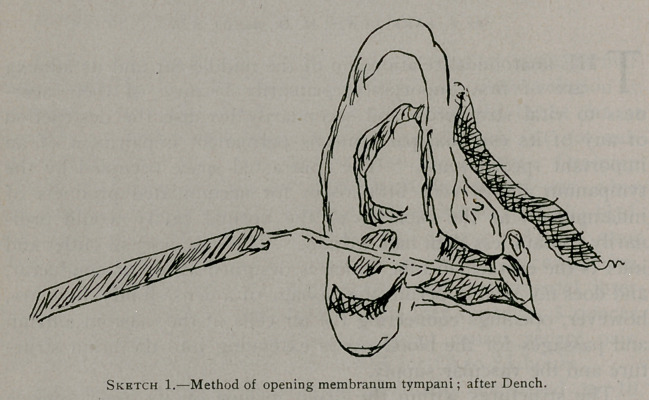


**Sketch 2. f2:**
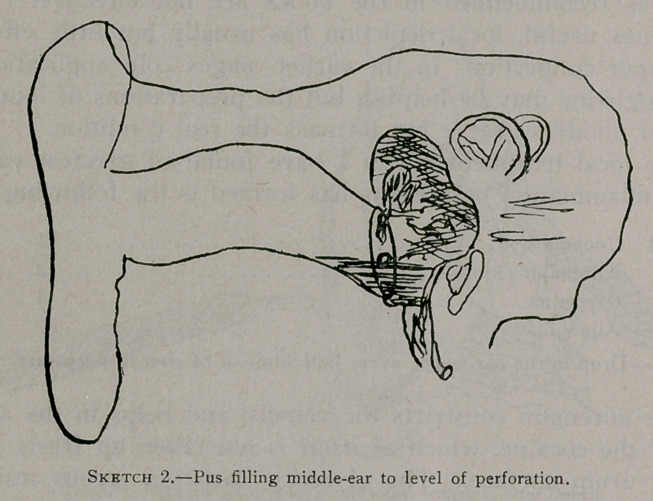


**Sketch 3. f3:**
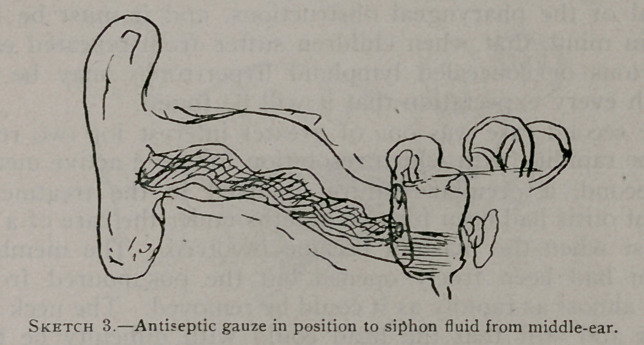


**Sketch 4. f4:**